# Rates of undiagnosed attention deficit hyperactivity disorder in London drug and alcohol detoxification units

**DOI:** 10.1186/1471-244X-12-223

**Published:** 2012-12-06

**Authors:** Zoe Huntley, Stefanos Maltezos, Charlotte Williams, Alun Morinan, Amy Hammon, David Ball, E Jane Marshall, Francis Keaney, Susan Young, Patrik Bolton, Karen Glaser, Raoul Howe-Forbes, Jonna Kuntsi, Kiriakos Xenitidis, Declan Murphy, Philip J Asherson

**Affiliations:** 1 MRC Social Genetic and Developmental Psychiatry, Institute of Psychiatry, Kings College London, London, SE 58AF, UK; 2 Behavioural Genetics Unit, Institute of Psychiatry, Kings College London, London, SE, 58AF, UK; 3 Addiction Unit, Maudsley Hospital, London, SE, 58AF, UK; 4 National Addiction Centre, Institute of Psychiatry, Kings College London and Bethlem Addiction Service, South London and Maudsley NHS Foundation Trust, London, SE, 5 8AF, UK; 5 Child and Adolescent Psychiatry, Institute of Psychiatry, Kings College London, London, SE, 58AF, UK; 6 Institute of Gerontology, King's College London, London, WC, 2R 2LS, UK; 7 Department of Forensic and Neurodevelopmental Sciences, Institute of Psychiatry, Kings College London, UK, SE58AF & Broadmoor Hospital, Crowthorne, Berkshire, UK, RG45 7EG

## Abstract

**Background:**

ADHD is a common childhood onset mental health disorder that persists into adulthood in two-thirds of cases. One of the most prevalent and impairing comorbidities of ADHD in adults are substance use disorders. We estimate rates of ADHD in patients with substance abuse disorders and delineate impairment in the co-morbid group.

**Method:**

Screening for ADHD followed by a research diagnostic interview in people attending in-patient drug and alcohol detoxification units.

**Results:**

We estimated prevalence of undiagnosed ADHD within substance use disorder in-patients in South London around 12%. Those individuals with substance use disorders and ADHD had significantly higher self-rated impairments across several domains of daily life; and higher rates of substance abuse and alcohol consumption, suicide attempts, and depression recorded in their case records.

**Conclusions:**

This study demonstrates the high rates of untreated ADHD within substance use disorder populations and the association of ADHD in such patients with greater levels of impairment. These are likely to be a source of additional impairment to patients and represent an increased burden on clinical services.

## Background

ADHD is a common childhood onset mental health disorder defined by the presence of impairing levels of hyperactive, impulsive and inattentive symptoms. The disorder has an estimated prevalence during childhood in the United Kingdom of 3.6% [1]. Longitudinal follow up studies find that around two-thirds of cases continue to be impaired by ADHD symptoms in adulthood, with an estimated worldwide prevalence for the disorder in adults of around 2.5% [2]. Despite the high rate of ADHD in adults, the disorder remains under-diagnosed and under-treated beyond the adolescent years [3]. This is a particular issue for adult mental health because ADHD symptoms lead not only to impairments in academic, occupational and social functioning, but are also associated with the development of comorbid disorders including anxiety, depression, personality disorder, antisocial behaviour and substance use disorders (SUD) [4-7]. This raises the question of whether people diagnosed with such conditions may in some cases have undiagnosed and untreated ADHD; and whether treatment of underlying ADHD may lead to improvements in their comorbid disorder. Among the most frequent comorbidities associated with ADHD are substance use disorders. A bi-directional link between ADHD and SUD has been reported, with increased rates of SUD within ADHD populations and increased rates of ADHD within SUD populations [8-10]. Previous studies of ADHD in the United States and Europe estimate very high prevalence rates for lifetime SUD of up to 58% [11]. These studies found that alcohol and cannabis are the most frequently abused substances [8] followed by cocaine and amphetamines [12]. There are potentially methodological issues that may inflate prevalence rates, indicated by the wide range in reported prevalence rates for ADHD in SUD populations of around 10% to 55% [13,14]. Challenges to the correct identification of ADHD in SUD settings include high drop-out rates between screening and assessment stages; and the potential overlap of symptoms between the two disorders [15] including intoxication and withdrawal states [12] and the long-term effects of chronic drug use on brain function [16-18] that could mimic ADHD. Retrospective recall of childhood ADHD symptoms is particularly challenging in SUD populations due to impaired memory and the often complex histories with adverse psychosocial risk factors for the development of behavioural problems. Informant reports are often not available in SUD populations because relationships with informants are frequently strained [12] and for this reason most studies depend on self-report. When evaluating ADHD, few papers report the time since last substance use, although delayed screening until three weeks of abstinence has been reported [19]. Lastly, it has been reported that autism is frequently unrecognized in people with ADHD and this may generate additional impairments in SUD populations.

This study addresses some of these concerns by implementing a systematic screening protocol for ADHD in London (UK) SUD clinics, with the aim of estimating the rate of undiagnosed and untreated ADHD within SUD patients; and evaluating whether those with comorbid ADHD and SUD experience greater impairment, in terms of co-morbid diagnoses, previous convictions or suicide attempts than those with SUD alone. We also carried out an investigation of autistic traits in this population to determine if this confounded our results.

## Methods

### Participants

#### Clinical samples

Sample characteristics are listed in Table 1. Participants (n=226) were recruited from two in-patient alcohol and drug detoxification and stabilisation units in South-East London. Consecutive admissions to the in-patient units were invited to take part in the study over an 18-month (Clinic 1) or 11-month (Clinic 2) period. Of these 74% had previously attended a detoxification treatment program. One participant already had a current adult diagnosis of ADHD and two had a recorded childhood diagnosis of ADHD. We excluded individuals with a history of current or recent psychosis (n=4), current serious physical illness (n=3), insufficient understanding of English to give informed consent (n=6) and lack of capacity to consent (n=2). Overall 51% of patients approached agreed to participate. Study participants received no payment for participation.

**Table 1:** Sample characteristics

**Total sample size**	**N=226**
Male gender	173 (76.5%)
Mean age*	39.0 (SD 10.3)
Ethnicity*	183 (81.0%) White European
Alcohol dependence*	112 (49.6%)
Drug dependence*	29 (12.8%)
Alcohol and drug dependence*	85 (37.6%)

### Study procedures

The study of people undergoing inpatient detoxification included two screening stages using ADHD rating scale data, followed by formal evaluation of the diagnosis of ADHD using a semi-structured interview for DSM-IV ADHD. By including two screening steps (T1 and T2) we were able to evaluate the difference between the level of ADHD symptoms on admission (T1) and one week later (T2), when they had been detoxified or stabilised on treatments such as methadone. Screening assessment session one (T1) took place soon after admission (mean day 5, SD 4). The second screening assessment session (T2) was completed as close as possible to seven days after the T1 assessment (mean 8 days, SD 6). During the T1 session, patients completed current and childhood symptom checklists for DSM-IV ADHD; and during the T2 session current symptoms only, since it was assumed that only the report of current levels of symptoms would be affected by state of detoxification and current drug use and needed to be measured at both time points. In addition whenever possible we obtained contact details from informants for childhood and current ADHD symptoms who were asked to complete an informant version of the screening questionnaire. Current informant ratings were obtained for 72 participants, and childhood ratings for 48 participants. The self-rated screening data were used to allocate participants into screen positive and negative groups for ADHD. Participants screening positive for ADHD were invited to complete a research diagnostic interview assessment. Participants who failed to attend the diagnostic interview were offered up to three further appointments. Following three missed appointments, a final letter invited participants to contact the research team to reschedule an appointment before the assessment was recorded as “missing”.

### Screening questionnaires

Participants completed DSM-IV 18-item self-report ADHD screening questionnaires for both current and childhood behaviour, using the 4-point (0, 1, 2, 3) rating scales from Barkley [20]. A symptom is considered present if the individual rates it as 2 (*often*) or 3 (*very often*). The Barkley rating scales offer a total symptom count for the inattentive domain and the hyperactive/impulsive domain (0–9 for each). We also asked participants to provide contact details for an informant to provide similar ratings for both current and childhood behaviour. Impairment questions from the Barkley scales [20] were included that asked participants to rate their level of impairment from ADHD symptoms on a four-point scale for 10 domains of function including: home life, work, social interactions, community activities, education, relationships, money management, driving, leisure activities and daily responsibilities. Participants also completed the Autism Quotient (AQ) [21] which screens for autism spectrum disorder (ASD). Participants with above threshold AQ scores (>31) were invited to complete an ADOS-G (Autism Diagnostic Observation Schedule-Generic) [22]. The ADOS-G algorithm scores participants as below threshold, autism spectrum or autism. Further objective information was collected from participants’ medical records, including details of current substance use and co-morbid diagnoses made by other mental health professionals. Previous convictions and previous suicide attempts were recorded as present or absent.

### Diagnostic interview

Research diagnosis of ADHD was established using the Diagnostic Interview for ADHD in Adults (DIVA 2.0; http://www.divacenter.eu) [23]), conducted by trained research assistants. All cases were reviewed by senior psychiatrists from the Maudsley Adult ADHD clinic (PA and SM). The interview systematically evaluates each of the DSM-IV symptom items for both current and childhood symptoms, and asks additional questions to establish impairment from ADHD symptoms (impairment criteria), in two or more settings (pervasiveness criteria) and the age of onset of symptoms (age of onset criteria). Reliability between interviewers over the first ten diagnostic interviews was high (Table 2).

**Table 2:** Inter-rater reliability for individual items on the diagnostic interview for ADHD in adults (DIVA)

		**Individual items (r)**	**4+ symptoms (CR)**	**6+ symptoms (CR)**
Reliability	Overall	0.875	98%	95%
	Child	0.900	95%	100%
	Adult	0.850	100%	90%

Intra-class correlations (r) for total symptom scores and concordance rates (CR) for diagnostic thresholds.

### Diagnostic algorithm

The diagnostic algorithm for ADHD used the following criteria: (i) 6 or more ADHD symptoms in either domain from retrospective childhood rating scales; (ii) 4 or more ADHD symptoms from either domain from current ADHD symptom rating scales; (iii) Research diagnosis of DSM-IV ADHD following application of diagnostic interview for DSM-IV ADHD. This study was approved by the South London and Maudsley Research Ethics Committee and all participants gave informed consent before taking part.

## Results

**Figure 1:**
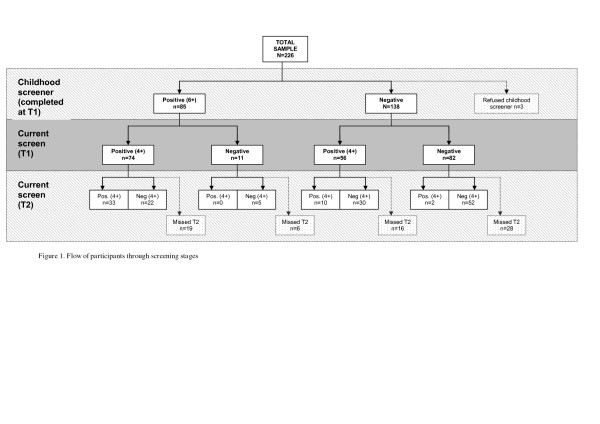
Flow of participants through screening stages.

Recruitment and screening stages are summarised in Figure 1. All 226 patients completed T1 screening questionnaires of which 69% completed T2 ratings. Reasons for missing T2 screeners included early self- or disciplinary-discharge (n=23), refusal to take further part in the study (n=15) and repeatedly missing T2 appointments (n=32).

### Childhood ADHD screeners

Mean total childhood ADHD symptom scores were highest in the group treated for drug dependency (mean 29, SD 14) followed by the combined drug and alcohol group (mean 24, SD 15) and the alcohol dependency group (mean 17, SD 14). A one way ANOVA showed significant effects of primary diagnosis (drug vs. alcohol vs. combined) on childhood screener total score (p<0.001). Planned contrasts showed significantly higher ratings of ADHD symptoms for drug compared to alcohol participants (p<0.001), but not between drug and combined drug and alcohol groups (p=0.12).

### T1 versus T2 ratings

These data are summarised in Table 3. Current ADHD scores dropped significantly between T1 and T2 ratings with a mean change of 8.6. We found that 52% of participants with T1 and T2 data had a clinically meaningful reduction of 8 or more points on the self-completed ADHD rating scales, which is equivalent to a one-level drop in the Clinical Global Impression Scale [24].

**Table 3:** Mean (SD) ADHD screening scores for retrospective ratings from childhood and current symptoms at T1 and T2 in the total inpatient sample

	**Childhood**	**T1**	**T2**	**Mean change T1 vs. T2**	**Paired t value (T1 vs T2 change)**
Total score /54	21.09	23.03	14.65	−8.6	11.92**
	(SD 15.0)	(SD 12.5)	(SD 12.1)	(SD 9.1)	
Inattentive symptoms /9	3.51	3.59	1.83	−1.80	10.21**
	(SD 3.2)	(SD 2.9)	(SD 2.5)	(SD 2.2)	
Hyp/imp symptoms /9	3.29	3.46	2.30	−1.23	7.40**
	(SD 3.0)	(SD 2.6)	(SD 2.6)	(SD 2.1)	

There is a significant reduction in ADHD symptoms following 1-week or more of treatment in the addiction units.

** Significant at p<0.001.

A 2x3 Mixed Model Factorial ANOVA on the time (T1 vs. T2) and primary diagnosis (drug vs. alcohol vs. combined drug and alcohol) found a significant main effect of time (p<0.001), indicating that self-rated ADHD symptom scores are influenced by the detoxification treatment process and/or withdrawal states following admission. A significant effect of primary diagnosis was also found (p<0.001) but no significant interaction between time and primary diagnosis (p=0.34). Between subjects contrasts showed significant differences for ADHD ratings between the alcohol and combined drug & alcohol groups (p<0.001), but not between drug and combined (p=0.28) or alcohol and drug (p=0.11), indicating higher levels of ADHD symptoms at T1 and T2 among those with drug abuse compared to those with alcohol abuse alone.

### Estimated prevalence of ADHD

**Figure 2:**
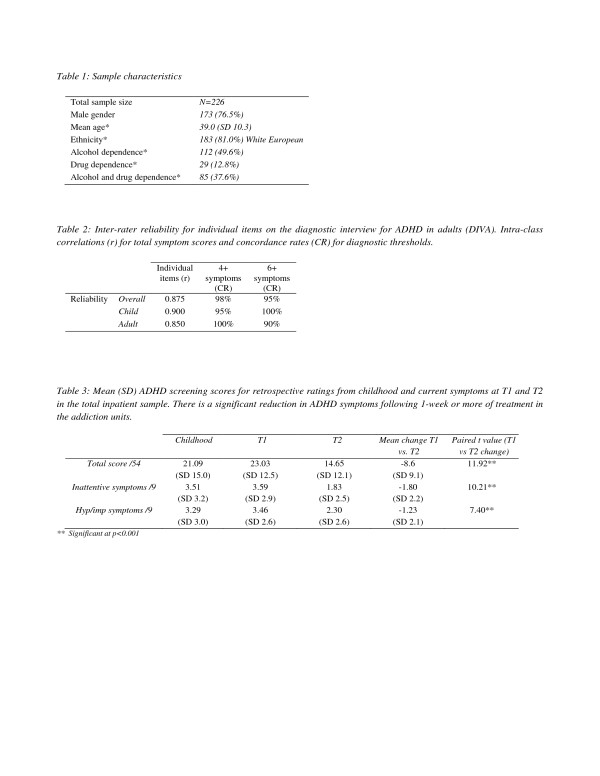
Estimated prevalence of ADHD.

There was an estimated prevalence rate for ADHD of 12.2% (Figure 2). Of these 73% met criteria for the combined subtype, 18% for the hyperactive-impulsive subtype and 9% for the inattentive subtype. The prevalence was calculated using the proportion of participants completing each step that did and did not fulfill ADHD screening and assessment criteria. This in turn allowed us to calculate an estimated prevalence rate of positive screens at each stage which is not affected by attrition rates between screening and assessment stages. To evaluate whether rates of ADHD might differ in the groups that screened positive for ADHD at T1 and T2, but did (or did not) attend for diagnostic interviews; we compared the attendees to the non-attendees. The participants who attended the diagnostic assessments showed no significant differences from those that did not at T1. However they had significantly higher score for inattentive (but not hyperactive-impulsive) symptoms at T2 (6.1 versus 4.3; p=0.025), suggesting that the prevalence estimate based on available data might be slightly inflated.

### Broader definitions of ADHD

Broadening the diagnostic threshold, based on the DIVA (diagnostic interview), only had a small impact on the prevalence estimate. For example reducing the threshold for diagnosing ADHD from ≥6 symptoms in childhood to ≥4 only increased the overall prevalence estimate to 14.4%; and no additional cases were identified by decreasing the threshold for current symptoms to ≥4.

### Informant rating scales

64% of participants provided contact details for informants of their current behaviour and 43% for childhood behaviour; and 50% of both sets of contacts returned screening questionnaires. The correlations between self and informant reported symptoms are listed in Table 4. Informant ratings of current ADHD symptoms correlated moderately with self-reported symptoms and were similar to the equivalent correlations in the control sample (r=0.47, p<0.001). However, the participants with positive screens for ADHD agreed far more strongly with their informants than those who did not screen positive for ADHD (correlations 0.62 to 0.66). Correlations between childhood self and informant ratings were similar to those for current behaviour but were non-significant. Overall, participants and informants were concordant for the 4+ screening criteria in 64% of cases at T1, 50% of cases at T2; and 81% concordance for the childhood 6+ criteria.

**Table 4:** Correlations between self and informant reports

		**Self report**	
		**T1**	**T2**
Current behaviour informant report	*All participants n=72*	.372**	.375*
	*Positive ADHD screen (childhood 6+ and T1 & T2 (if applicable) 4+) n=16*	.617*	.654*
	*Negative ADHD screeners n=56*	.258	.169
		Childhood screener	
Childhood behaviour informant report	*All participants n=47*	.391**	
	*Positive ADHD screeners (childhood 6+) n=20*	.231	
	*Negative ADHD screeners n=27*	.293	

** Significant at p<0.01 / *Significant at p<0.05.

### Impairment & substance use

**Figure 3:**
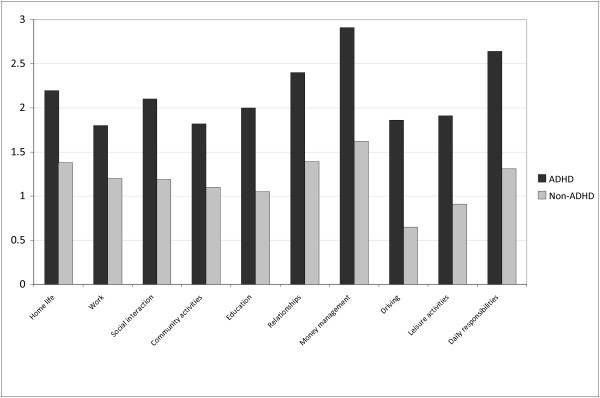
Mean impairment scores at T1 (/3) (inpatient sample).

As expected the SUD sample rated themselves as significantly more impaired than controls (p<0.05). Among the individuals with SUD there was a significant positive relationship between self-rated impairment and the severity of ADHD symptoms in childhood and at both T1 and T2 (respectively: r=0.52, p<0.001; r=0.73, p<0.001; r=0.51, p<0.001). Furthermore, greater impairment was significantly negatively correlated with change scores between T1 and T2 (r=−0.23, p<0.01); indicating that participants with greater impairment at T1 showed a smaller drop in ADHD symptoms between T1 and T2. Lastly, within the SUD population we compared the diagnosed ADHD cases to the non ADHD cases. Those with ADHD had significantly higher rates of self-reported impairments (see Figure 3) and were significantly more likely to have reported using cocaine or amphetamines (Table 5), consumed more units of alcohol per day and had more prior suicide attempts. Also they had a trend for higher rates of depression, previous convictions and previous drug or alcohol dependency treatment (Table 6).

**Table 5:** Drug use (past or current) in ADHD vs. non-ADHD groups

	**Heroin**	**Amphetamine**	**Crack cocaine**	**Cocaine**	**Marijuana**
ADHD (n=11)	45.5%	63.7%	18.2%	54.6%	36.4%
Non-ADHD (n=182)	29.7%	6.6%	29.1%	11.0%	15.4%
Likelihood Ratio	1.141	21.289	.663	11.361	2.685
p value	.285	<0.001**	.416	.001**	.101

** Significant at p<0.01.

**Table 6:** Other indicators of impairment in ADHD vs. non-ADHD groups

	**Previous suicide attempts**	**Comorbid depression diagnosis**
ADHD (n=11)	54.5%	27.3%
Non-ADHD (n=183)	23.0%	35.0%
Likelihood Ratio	4.675	5.714
p value	.029*	.057

*Significant at p<0.05.

### Autism spectrum disorder

Only 4 participants out of 113 who completed the AQ were above the threshold and of these only 1 met criteria for possible ASD. Since the rates of ASD in this population, based initially on self-report data, appear to be very low, we did not pursue this line of investigation further. Future studies should consider informant reports of autistic behaviours at the screening stage, to exclude the possibility of low reporting of ASD behaviours using self-report questionnaires.

## Discussion

Our main finding was that the estimated prevalence of undiagnosed ADHD within substance use disorder clinic populations in South London is around 12%. This is of importance as we also found evidence that those individuals with both SUD and ADHD had significantly higher self-rated impairments across several domains of daily life; and higher rates of substance abuse and alcohol consumption, suicide attempts and depression, recorded in their case notes. Taken together these two findings highlight the negative impact of ADHD on the individual and the increased burden they most likely place on services. When considering the generalisablity of these findings, a limitation of this study is the focus on in-patient detoxification units in London. Further work is therefore required to evaluate rates and impact of ADHD among out-patient SUD populations and other national and international regions.

### Impairments associated with comorbid ADHD and SUD

The finding of higher levels of impairment for ADHD cases within the SUD sample appears to be robust since we found indicators of impairment from both self-report and more objective (case note) measures. Appropriate assessment and management of ADHD in SUD patients would therefore seem to be potentially important to improve the general level of functional impairments and, particularly, given our finding that ADHD is associated with increased frequency of suicide attempts and depression. Whether treating ADHD in the context of SUD improves depression has yet to be adequately studied; yet we know that in people with ADHD symptoms such as mood instability and low self-esteem respond well to treatments for ADHD [25-28]. Another finding was that the ADHD group had significantly higher use of stimulants such as cocaine and amphetamine (although not crack cocaine). One possible (but untested) explanation for this, given that stimulants are routinely used to treat ADHD in the general population, is that people with ADHD are using stimulants as a form of self-treatment. However an additional mechanism could be that people with ADHD have a preference for drugs that are more ‘stimulating’, especially when injected or taken at high dose. Further work is needed to address this issue.

### Definition of ADHD and methodological issues

Our work attempted to address, as best we could, the inevitable methodological difficulties that might impact on accurate estimation of prevalence rates for ADHD within people with SUD. These include the potential for unreliable information from self-report questionnaires, difficulties with retrospective recall of childhood symptoms and the direct impact of drug and alcohol intoxication and withdrawal states on ADHD-like symptoms. We therefore attempted to measure ADHD symptoms before and after detoxification, included independent informant ratings whenever possible, compared these to population control data, and completed research diagnostic interviews. One additional confounder might arise if the study participants considered a positive diagnosis of ADHD as a way to obtain stimulant medication. However the study was not linked directly to the treatment of ADHD and expectations for treatment with stimulants are currently low in the UK, because in most cases ADHD in adults goes unrecognised and untreated [3]. Based on the percentage of patients who screened positive for ADHD in childhood and adulthood and the proportion of those interviewed we estimated an overall rate of ADHD of around 12% in our sample, much higher than the equivalent estimated worldwide prevalence of around 2.5% to 3.4% in non SUD populations [2,4,29]. Using slightly broader criteria for ADHD to reflect different approaches to the diagnosis of adult ADHD being taken by different investigators and clinicians, we found the estimated prevalence in our sample increased to a maximum of 15%. Overall, the estimated prevalence of ADHD in our sample is far higher than population rates, yet lower than those cited in some previous studies of ADHD in SUD populations. There are several potential reasons for these differences in the findings from this and previous studies. One potential reason was our stringent application of the DSM-IV criteria, which might have led to an underestimate of the true rate of ADHD in the SUD population for several reasons. First, the inherent problem in collecting childhood data retrospectively might mean that some participants who met current criteria for ADHD may have been unable to recall sufficient examples of childhood symptoms. We recorded ‘unknown’ ratings in the diagnostic interview assessments if participants could not provide sufficient information to conclude that a symptom was present or absent and found that on average 15% of childhood symptoms could not be scored. Secondly, DSM-IV criteria only require that ‘some’ symptoms and impairments were present during early childhood and do not specifically require ≥6 symptoms from childhood so long at the symptom count criteria are currently met as an adult. Nevertheless, we decided to take the more stringent approach because of the lack of prospective data from childhood and to guard against inclusion of ADHD-like syndromes that might arise as a result of chronic drug abuse in the absence of an underlying ADHD diagnosis. Finally, we know that the current DSM-IV criterion are not adjusted to take into account age-related changes in the development of ADHD and there is evidence that ≥ 4 symptoms in adults, rather than the current ≥6 symptoms, is sufficient; indeed this change is being considered for the fifth revision of the DSM that is currently in preparation [3,30,31]. However, taking all these alternative thresholds into account had only a minor impact on our estimate of the prevalence of ADHD in the SUD population.

### The impact of drug detoxification on ADHD symptoms

We investigated the impact of drug intoxication and/or the detoxification process by evaluating self-rated ADHD symptom scores a few days after admission to the detoxification unit and one week later, when they were detoxified or stabilized on long term medication and no longer in a withdrawal state. We found significant decreases in ADHD symptoms of around 8-points (15% of the total score), which is a clinically significant reduction in ADHD symptoms and comparable to a one-level drop of the Clinical Global Impression Scale [24]. In terms of our screening criteria this led to 40% of patients no longer meeting screening criteria for ADHD at T2 compared to the T1. Hence prior studies may have reported a higher prevalence than we found due to the confounding effect of drug use and/or withdrawal symptoms. Other researchers have noted mood disturbances during alcohol detoxification [32] and it is therefore possible that ADHD-like symptoms are also part of the withdrawal syndrome. Despite this, we found that in 60% of cases self-rated ADHD symptoms remained clinically significant following completion of the detoxification process. One implication is that although the withdrawal process may impact on the level of ADHD symptoms (and this should be taken into account when evaluating ADHD in SUD patients) there still remain a significant number of individuals with clinically significant symptoms of ADHD – and these require treatment.

### Clinical implications

Our findings suggest that clinical evaluations for ADHD are probably best completed once detoxification or stabilization for drug or alcohol dependency has been completed. However this suggestion may be difficult to implement in community patients. Furthermore, once diagnosis of ADHD has been established it will be important to offer treatment. Use of some pharmacological treatments (such as use of stimulants) is complex, however, in those with a current SUD. Nevertheless therapeutic nihilism is not an option as treatment of underlying ADHD may be important to the success of drug treatment programs for some individuals. It might be advisable to use the more stringent screening criteria of 6+ symptoms in either domain to identify those that need full clinical evaluation for ADHD, or to recognise the need for detailed ADHD assessment in a higher proportion of cases. Moreover it may be more appropriate to use non stimulant medications such as atomoxetine, as a first line treatment. Further work is required to address this issue, and the potential for using CBT based therapies for ADHD in this complex population. Obtaining informant data on ADHD symptoms for childhood and for current symptoms proved difficult in this population. The reasons for this were not investigated here, but likely reflect the often poor relationships that many SUD patients have with their family and friends. Therefore in clinical practice it will also often be the case that the diagnostic assessment of ADHD will depend on self-report alone. We were able to investigate the validity of these self-reported data by comparison of self-report with informant reported data in a subset of our sample and a comparison control sample. These showed moderate correlations between raters which were similar to that seen in non-SUD control populations. For the most impaired sub-group however, who were screening positive for ADHD based on their self-report, there was a far higher correlation with informant report for current ADHD ratings of around 0.62–0.65. We therefore suggest that while discrepancies between raters exist for ADHD rating scales, this does not appear to be different for SUD compared to control populations. Furthermore ratings showed moderately high levels of agreement for the group of patients with ADHD. Previous research has shown that in general people with ADHD tend to rate their symptoms lower than informants [20] perhaps reflecting difficulties in self-evaluation of ADHD symptoms. Our research does not support this finding within the SUD population investigated here. When we completed diagnostic interviews with 26 people that screened positive for ADHD on the basis of their self-rated symptoms we found that only 61% met full criteria for ADHD. The clinical implication of this finding is that the diagnosis of ADHD in patients with SUD should not depend solely on rating scale data, but rather on the basis of more objective examples of symptoms characteristic of ADHD, as applied here using the DIVA interview. Rating scales are a valuable tool for screening for ADHD but should not be used as a replacement for a full diagnostic assessment by clinical interview. Furthermore, while informant reports are helpful in supporting the diagnosis, the moderately high correlations with self-report suggest that in most cases self-report alone should be sufficient. For in-patient SUD units it should also be feasible to observe patients for level of restlessness, problems with self-organisation, inattentiveness, impulsive responses and poor emotional regulation, that are characteristic of ADHD in adults.

## Conclusions

This study applied stringent ADHD diagnostic criteria, necessary to avoid mistaking withdrawal states and other mental health problems for ADHD. This resulted in identification of a relatively small ADHD group compared to other studies relying only on self-report screening questionnaires. Despite this, our findings confirm high rates of ADHD within SUD populations that are approximately 5-fold higher than general population rates. Furthermore SUD patients with high levels of ADHD were functionally more impaired (including higher rates of suicide attempts). This study highlights the importance of identifying the sub-group of people with both SUD and ADHD. Further studies are required to evaluate the effectiveness of targeted treatments for ADHD within SUD patient populations. 

## Competing interests

The following co-authors have no conflicts of interest: Karen Glaser, Patrick Bolton, Francis Keaney, Alun Morinan, Lotte Williams, Stefanos Maltezos, Zoe Huntley, Amy Brinsford, David Ball, Raoul Howe-Forbes, Kiriakos Xentidis and Jane Marshall. The following co-authors have the following conflict of interest: Philip Asherson has received funds on behalf of Kings College London for advisory board and/or consultancy for Janssen-Cilag, Shire, Flynn Pharma and Eli-Lilly. He received educational and research grants from Janssen, Vifor, Shire and QbTech. Declan Murphy is currently funded by MRC, Wellcome trust and the Thomas Bailey Foundation. Jonna Kuntsi has received a speaker’s fee from Eli Lilly that has been used for educational and research activities and is funed by Action Medical Research. Susan Young has been a consultant for Janssen-Cilag, Eli-Lilly and Shire. She has given educational talks at meetings sponsored by Janssen-Cilag, Shire, Novartis, Eli-Lilly and Flynn-Pharma and has received research grants from Janssen-Cilag, Eli-Lilly and Shire.

## Authors’ contributions

PA, DM, PB and KG obtained funding and conceived of the project and study design with SY. Other co-authors were involved in coordination and collection of data at the clinical sites. Analyses completed by ZH. PA and ZH wrote the first and final drafts with review and comments from all authors listed. All authors read and approved the final manuscript.

## Pre-publication history

The pre-publication history for this paper can be accessed here:

http://www.biomedcentral.com/1471-244X/12/223/prepub
